# Strategies to work with HLA data in human populations for histocompatibility, clinical transplantation, epidemiology and population genetics: HLA-NET methodological recommendations

**DOI:** 10.1111/j.1744-313X.2012.01113.x

**Published:** 2012-12

**Authors:** A Sanchez-Mazas, B Vidan-Jeras, J M Nunes, G Fischer, A-M Little, U Bekmane, S Buhler, S Buus, F H J Claas, A Dormoy, V Dubois, E Eglite, J F Eliaou, F Gonzalez-Galarza, Z Grubic, M Ivanova, B Lie, D Ligeiro, M L Lokki, B Martins da Silva, J Martorell, D Mendonça, D Middleton, D Papioannou Voniatis, C Papasteriades, F Poli, M E Riccio, M Spyropoulou Vlachou, G Sulcebe, S Tonks, M Toungouz Nevessignsky, C Vangenot, A-M van Walraven, J-M Tiercy

**Affiliations:** 1 University of GenevaGeneva, Switzerland; 2 Blood Transfusion Centre of SloveniaLjubljana, Slovenia; 3 Medical University of ViennaVienna, Austria; 4 Gartnavel General HospitalGlasgow, UK; 5 Riga Stradins UniversityRiga, Latvia; 6 University of CopenhagenCopenhagen, Denmark; 7 Leiden University Medical CenterLeiden, The Netherlands; 8 EFSStrasbourg, France; 9 EFS Rhone AlpesLyon, France; 10 Saint-Eloi Hospital, MontpellierFrance; 11 University of LiverpoolLiverpool, United Kingdom (Great Britain); 12 University Hospital ZagrebZagreb, Croatia; 13 University Hospital AlexandrovskaSofia, Bulgaria; 14 Rikshospitalet, Oslo University HospitalOslo, Norway; 15 Centro de Histocompatibilidad do SulLisboa, Portugal; 16 University of HelsinkiHelsinki, Finland; 17 University of PortoPorto, Portugal; 18 H. Clinic de BarcelonaBarcelona, Spain; 19 Transplant Immunology Laboratory, Royal Liverpool and Broadgreen University HospitalUK; 20 Ministry of HealthNicosia, Cyprus; 21 Evangelismos General Hospital of AthensAthens, Greece; 22 Ospedale Maggiore PoliclinicoMilano, Italy; 23 General University Hospital of Athens AlexandraAthens, Greece; 24 University Hospital CenterTirana, Albania; 25 University of OxfordOxford, UK; 26 Erasme HospitalBrussels, Belgium; 27 University Hospital GenevaGeneva, Switzerland

## Abstract

HLA-NET (a European COST Action) aims at networking researchers working in bone marrow transplantation, epidemiology and population genetics to improve the molecular characterization of the HLA genetic diversity of human populations, with an expected strong impact on both public health and fundamental research. Such improvements involve finding consensual strategies to characterize human populations and samples and report HLA molecular typings and ambiguities; proposing user-friendly access to databases and computer tools and defining minimal requirements related to ethical aspects. The overall outcome is the provision of population genetic characterizations and comparisons in a standard way by all interested laboratories. This article reports the recommendations of four working groups (WG1-4) of the HLA-NET network at the mid-term of its activities. WG1 (Population definitions and sampling strategies for population genetics’ analyses) recommends avoiding outdated racial classifications and population names (e.g. ‘Caucasian’) and using instead geographic and/or cultural (e.g. linguistic) criteria to describe human populations (e.g. ‘pan-European’). A standard ‘HLA-NET POPULATION DATA QUESTIONNAIRE’ has been finalized and is available for the whole HLA community. WG2 (HLA typing standards for population genetics analyses) recommends retaining maximal information when reporting HLA typing results. Rather than using the National Marrow Donor Program coding system, all ambiguities should be provided by listing all allele pairs required to explain each genotype, according to the formats proposed in ‘HLA-NET GUIDELINES FOR REPORTING HLA TYPINGS’. The group also suggests taking into account a preliminary list of alleles defined by polymorphisms outside the peptide-binding sites that may affect population genetic statistics because of significant frequencies. WG3 (Bioinformatic strategies for HLA population data storage and analysis) recommends the use of programs capable of dealing with ambiguous data, such as the ‘gene[rate]’ computer tools to estimate frequencies, test for Hardy–Weinberg equilibrium and selective neutrality on data containing any number and kind of ambiguities. WG4 (Ethical issues) proposes to adopt thorough general principles for any HLA population study to ensure that it conforms to (inter)national legislation or recommendations/guidelines. All HLA-NET guidelines and tools are available through its website http://hla-net.eu.

## Introduction

Our knowledge of the genetic diversity of the human species has expanded considerably in recent decades, thanks to the rapid progress in genomic research. The possibility of genotyping individuals at high resolution over the entire genome (Altshuler *et al.*, 2010), and specifically the Major Histocompatibility Complex (MHC), through a thorough characterization of DNA sequence variation at human leukocyte antigen (HLA) genes (The MHC sequencing consortium., 1999; Robinson *et al.*, 2003) has been crucial in addressing major issues related to biomedicine and molecular biosciences, such as the assessment of genetic susceptibilities to diseases (Segal & Hill, 2003; de Bakker *et al.*, 2006), the control of haematopoieic stem cell and organ transplantation (Hansen *et al.*, 1999; Petersdorf *et al.*, 2003; Mehra, 2010), the appraisal of the genetic structure of human populations and its meaning (Buhler & Sanchez-Mazas, 2011) and the understanding of genomic evolution in relation to the environment (Meyer & Thomson, 2001, for a review; Sanchez-Mazas *et al.*, 2012), among other essential topics. A common goal of these studies is to estimate HLA genetic diversity within and among human populations and to describe it through the molecular typing of population samples.

In this context, a main challenge of tissue typing (or histocompatibility) laboratories involved in clinical research (donor–recipient matching) is to produce HLA molecular data of high quality. Such laboratories address a crucial health problem in modern societies, the need for haematopoietic stem cell transplantation involving the search for HLA compatible donors. Haematopoietic stem cell volunteer donors are generally recruited randomly in each country with an effort to constitute very large registries reflecting the HLA variation over different regions (e.g. Kollman *et al.*, 2004; Schmidt *et al.*, 2010). In addition, some registries specifically aim to improve recruitment from ethnic minorities (e.g. Johansen *et al.*, 2008) to increase the HLA diversity and hence the probability of finding an appropriate donor for a given patient. In this context, knowledge of the distribution of alleles and haplotypes in many different population groups as determined by high-resolution typing may allow the design of more efficient recruitment algorithms.

The accurate description of allelic and haplotypic HLA profiles and the identification of rare HLA variants in human populations is not only crucial to recipient–donor matching and research projects on histocompatibility. In addition, researchers in at least two other disciplines share related objectives. Firstly, as HLA genes play an essential role in susceptibility or resistance to serious human diseases (Svejgaard *et al.*, 1996; Blackwell *et al.*, 2009), such as HIV (Carrington *et al.*, 1999; Kawashima *et al.*, 2009; Pereyra *et al.*, 2010), their meticulous molecular analysis underpins epidemiological research. Statistically reliable comparisons between case and control population samples are needed to assess the susceptibility (or resistance) conferred by specific HLA alleles. Knowledge of the prevalence of a susceptibility allele in a given population is crucial to evaluate the genetic risk provided by several different HLA alleles in autoimmunity, infectious diseases or allergic reactions to drugs.

Secondly, HLA genes are of particular interest from a population genetics point of view to study the genetic history of the human species and the mechanisms of molecular evolution (Meyer *et al.*, 2006; Buhler & Sanchez-Mazas, 2011). Different human populations exhibit different HLA genetic profiles. This is partly explained by the geographic dispersal of modern humans throughout the world and partly by an effect of natural selection (Meyer *et al.*, 2006; Solberg *et al.*, 2008; Buhler & Sanchez-Mazas, 2011; Sanchez-Mazas *et al.*, 2011). Indeed, the evolution of HLA may be driven by an advantage of specific alleles but also by an advantage conferred to heterozygous individuals against a large variety of pathogens (Prugnolle *et al.*, 2005; Sanchez-Mazas *et al.*, 2012). A precise knowledge of the distribution of allele frequencies in many different populations may help to understand human peopling history and the interaction of populations with their environment in a pathogenic context.

HLA-NET (http://hla-net.eu), a European network of laboratories involved in the study of HLA for histocompatibility, epidemiology and/or population genetics, was created in 2009 to achieve highly significant goals in the present research context. Despite their different objectives and applications, all laboratories involved in this network are united by a common research task, the description of HLA molecular diversity in human populations, to get accurate reference data for their own studies in different disciplines and to provide pan-European data to research groups working internationally. Moreover, these laboratories are concerned with similar types of methodological problems raised by the complexity of HLA polymorphism, i.e. how can a population sample for different applications be defined accurately? How can data be generated that are comparable to those of other laboratories? How can gene frequencies and other statistics with highly complex data be estimated? What legal and ethical rules should be followed to harmonize with national requirements? HLA-NET is designed to answer those questions via standardization of protocols and procedures and the development of an electronic platform to collect, handle, store and process HLA data and share information amongst European laboratories. Its final objective is to improve qualitatively and quantitatively the collection of HLA-typed population samples all over Europe and surrounding areas and to produce a consensual map of HLA molecular diversity for this broad geographic region.

This article reports the achievements and provides the main recommendations of HLA-NET at the mid-term of its activities. The results are presented by four working groups (WGs) addressing crucial questions related to the main issues mentioned above: population definitions and sampling strategies for population genetics analyses (WG1), HLA typing standards for population genetics analyses (WG2), bioinformatics strategies for HLA population data storage and analysis (WG3) and ethical issues (WG4). A list of laboratories contributing to the HLA-NET project is also presented.

## WG1 – Population definitions and sampling strategies for population genetics’ analyses

### Aims of group

Working group 1 (WG1) aims at improving the quality of population data used in HLA-related studies in terms of population definition and sampling and at coordinating the collection of HLA-typed population samples from Europe and surrounding areas.

#### Population definition and sampling

The establishment of standardized procedures and questionnaires for collecting and databasing HLA-typed population samples is essential to fill in the current lack of comparability among different studies pursuing similar goals: a reliable estimation of HLA gene frequencies in samples of healthy individuals to compare with patients suffering from severe diseases (HBV, HIV, rheumatoid arthritis, etc.), a reliable estimation of HLA gene frequencies in ethnically or geographically well-defined populations to reconstruct human peopling history or a reliable identification of rare HLA alleles or multilocus haplotypes in distinct populations to optimize the search of potential donors in haematopoietic stem cell transplantation.

An important issue is the definition of populations from an ‘anthropological’ point of view. The group decided to avoid *a priori* misclassifications of racial and ethnic groups in both questionnaires and databases and to consider several levels of description related to geographic origin, language(s) spoken and any other relevant information on the ancestry of each studied population. Outdated racial or ethnic definitions like ‘Caucasian’ are to be replaced with ethically acceptable alternative names.

##### ‘Caucasian’: a meaningless definition

HLA-NET recommends avoiding the term ‘Caucasian’, as well as its derivatives ‘Caucasoid’ and related terms. To understand the reasons of this recommendation, one has to bear in mind the complex history of European populations and their present biological and cultural diversity.

There have been difficult discussions among geneticists on the proportion of Palaeolithic, Mesolithic or Neolithic ancestry of European populations going back to very different periods of time (some 40 000, 18 000 or 10 000 years ago, respectively) (e.g. Balaresque *et al.*, 2010; Chikhi *et al.*, 1998, 2002; Pereira *et al.*, 2005; Richards *et al.*, 2000; Semino *et al.*, 2000), and such controversies have also been raised by analyses on ancient DNA (Ammerman *et al.*, 2006; Barbujani & Chikhi, 2006). Even the proportion of Neandertal contribution to the genetic pool of modern Europeans is currently disputed, ranging from no contribution to around 4% of interbreeding between Neandertals and modern humans (Currat & Excoffier, 2004, 2011; Serre *et al.*, 2004; Green *et al.*, 2010). Although genetic studies do not yet provide firm conclusions to these issues, archaeological data show that the migrations of Neolithic farmers from the Near East led to major transformations in diverse aspects of European life styles (Tresset & Vigne, 2011). Also, the significant HLA genetic structure observed in present-day Europeans may possibly trace back to that period (Buhler *et al.*, 2006).

Europe has been subjected to heterogeneous climates in the past and is nowadays characterized by temperate to cold temperatures, marked seasons and highly variable environments. Present-day Europeans are characterized by a huge phenotypic diversity with pronounced differences, for example, in hair and eye colour and body height (with small and tall populations). Even skin colour varies from relatively dark in some southern populations to very light in the north. Such phenotypes were most probably shaped by adaptive selection to different environments (Sabeti *et al.*, 2007; Sturm, 2009) although the intensity of selection may have varied greatly among different traits. Some other phenotypic traits, which are not visible to the naked eye because they concern specific molecules involved in internal metabolic pathways, exhibit unusual patterns in Europe. This is the case for lactase persistence: most southern Europeans cannot digest milk in adulthood (like most people in the world) while northern Europeans are perfectly adapted to milk consumption, and this is because of loss of activity of the lactase enzyme after weaning in the former (Ingram *et al.*, 2009). This trait has evolved partly through natural selection, in coevolution with animal domestication and/or through an effect of climate, and partly as a consequence of the demographic expansions occurring during the Neolithic period (Gerbault *et al.*, 2009, 2011). It also illustrates the high level of genetic diversity of European populations, with a frequency of the lactase persistence allele varying from 0 to almost 80% from south to north.

Europe also exhibits a high cultural complexity, reflected, for example, by the diversity of the languages that are spoken today in this continent. There are almost 50 languages belonging to a dozen families, some of which belong to unrelated linguistic phyla including Indo-European, Uralic and Basque (http://ethnologue.com). The origin of this diversity is not yet fully understood: for example, there are competing theories on the origin of Indo-Europeans (do they come from the Near-East or from the north of the Black Sea, or both?) (Diamond & Bellwood, 2003; Gray & Atkinson, 2003; Balter, 2004) and the origin of some isolated populations, such as Basques, is still uncertain.

The history of Europe and its surrounding areas is so complex and its population diversity so high that the use of a unique term, ‘Caucasian’, to describe all populations from Europe and its surrounding areas is a crude simplification, which is clearly not appropriate. Actually, the term ‘Caucasian’ was first used by the German naturalist Johann Friedrich Blumenbach at the end of the 18th century (Gould, 1996). Blumenbach, during his journeys, found that the people, and more particularly the women, living in the Caucasus were exceptionally wonderful. In his famous book on *The unity of the human genus and its varieties* published in 1795, he thus described the European variety as the ‘Caucasian’ variety. Later on, the term ‘Caucasian’ (or its derivatives ‘Caucasoid’, ‘Aryan’, etc.) remained in the anthropological classifications to describe a prototype of Europeans (obviously influenced by a racist ideology, with dramatic consequences during world history).

For such reasons, the terms ‘Caucasian’ and its derivatives have to be deleted from the scientific vocabulary. HLA-NET proposes to replace them by the following substitutes, depending on each specific situation:

1. ‘*Europeans*’, for populations of European origin living in Europe;
2. ‘*populations of European descent*’, for populations of European origin not living in Europe;
3. ‘*populations from* (where they are from) *living in Europe*’, for populations of non-European origin living in (where they live) in Europe;
4. ‘*North Africans*’, ‘*West Asians*’, ‘*populations from the Near East*’ and other geographic names when populations from these areas surrounding Europe are concerned;
5. ‘*pan-Europeans*’, if a general expression is needed to name at the same time the populations from Europe and those from its surrounding areas North Africa, the Near East and Western Asia.

##### ‘Black’, ‘Mongoloid’ and other outdated and connoted terms

Because HLA-NET is a European Action focusing on the HLA molecular characterization of pan-European populations, we concentrated our discussion above on the biological and cultural diversity of Europeans and the misuse of the term ‘*Caucasian*’. However, our network is also aware that other outdated terms are commonly used to name groups of populations from other continents and recommends avoiding them:

‘Black’ or ‘African Black’ (or even ‘Negroid’) are terms inherited from several centuries (18^th^ to 20th) of colonial (and racist) anthropology (see, for example, *The Outline of History of Mankind*, by polygenist Christoph Meiners, published in German in 1785). Nevertheless, they are still frequently used by researchers to name sub-Saharan Africans, because of the generally very dark skin of these populations. Here again, time has come to definitively abandon such appellations, which do not correspond to any scientific classification. Sub-Saharan African populations are highly diverse from a biological point of view, both in terms of genetic variation (as most genetic studies have largely demonstrated) and variation of some quantitative traits including, for example, cranial measurements (Relethford & Harpending, 1994) and hair shape (De la Mettrie *et al.*, 2007). Although skin colour may also vary significantly in sub-Saharan Africa (e.g. between East and South Africans, Khoisan, Pygmies, etc.), this trait has followed a more peculiar evolution which has been strongly governed by latitude-dependent natural selection (see, for example, Parra, 2007; and Rees & Harding, 2012), explaining its unusual diversity pattern throughout the world (Relethford & Harpending, 1994). As a result, very dark-skinned people exist in all continents, from Africa to Australia via India, Southeast Asia and Melanesia. Taking language as a cultural marker, Africa is also highly diverse from a cultural point of view, grouping 30.5% of the total world languages (http://www.ethnologue.com) and four main linguistic phyla, the dispersal of which reveals a complex history of this continent (Excoffier *et al.*, 1987; Blench, 2006). Similar to ‘Caucasian’, HLA-NET thus recommends using terms other than ‘Black Africans’ and derivatives, such as:

1. ‘*sub-Saharan Africans*’, for populations of African origin living south of the Saharan Desert;
2. ‘*North Africans*’, for populations of African origin living north of the Saharan Desert;
3. ‘*West Africans*’, ‘*South Africans*’, ‘*East Africans*’, or even more detailed geographic names, for populations of African origin living in the respective geographic areas;
4. ‘*populations of African descent*’, for populations of African origin not living in Africa.

‘Mongoloid’ is also used today in anthropology, although less frequently than ‘Caucasian’ and ‘Black African’. It is based on apparent similarities of phenotypic traits (such as the epicanthic fold of the eye, very pronounced in populations from Mongolia) between all Asian populations, just as ‘Black’ refers to skin colour resemblances. Like ‘Caucasian’, ‘Mongolian’, which is actually correct to name the inhabitants of Mongolia, but not to name a human race, was used by Meiners and Blumenbach in their racial classifications. Both ‘Mongoloid’ and ‘Mongolian’ (taken in that sense) are again unfortunate relics of the reductionist views on human variation prevailing in the last centuries. In agreement with the most commonly used expressions today, we thus propose to replace these terms and their derivatives by the following appellations:

1. ‘*Asians*’, for populations of Asian origin living in Asia;
2. ‘*West Asians*’, ‘*South Asians*’, ‘*East Asians*’, ‘*Southeast Asians*’, ‘*Northeast Asians*’, or even more detailed geographic names, for populations of Asian origin living in the respective geographic areas;
3. ‘*populations of Asian descent*’, for populations of Asian origin not living in Asia.

##### HLA-NET population data questionnaire

WG1 has worked on a standard questionnaire to characterize populations and the population samples collected for HLA typing, which have to be representative and statistically reliable. This questionnaire is available at http://hla-net.eu/population_questionnaire and shown in Appendix 1. Note that it has been used as a standard document for *AHPD* (Analysis of HLA Population Data), a project of the 16th International Histocompatibility and Immunogenetics Workshop (IHIW).

Basically, one has to provide, for each sample tested (or to be tested) for HLA:

1. The type of study (i.e. origin of the sample): in principle, the population samples of interest for this project are to be defined on specific criteria based on anthropological field studies (see below points 1–4); however, for statistical reasons related to the number of available samples and of individuals per sample, bone marrow registry data can also be considered and used under clear-cut conditions. Also, collection of patients, although generally not used for studies in anthropology, may be useful at a later stage if a specific epidemiological project is undertaken. They are thus not excluded *a priori*. The information on the type of study is important to know whether a given sample may include individuals of diverse origins or who share some peculiar characteristics (e.g. to suffer from a given disease). Any deviation from Hardy–Weinberg equilibrium or other unexpected result may then be better understood. Other important information is the presence of close (first-degree) relatives in the sample, as this may impair the estimation of gene frequencies and Hardy–Weinberg equilibrium. The inclusion of more remote relatives (cousins, etc.) may also introduce some bias but cannot be avoided, in particular if samples are taken from isolated populations studied in the field, which are often highly endogamous. This is why we only require *a priori* the exclusion of first-degree relatives.
2. The name of the population represented by the sample: we propose the *Ethnologue* as an excellent guide to find consensual and alternative names of the populations under study (although these are linguistic names, they most often correspond to the used ethnic names). Some alternative names (e.g. names given by the population to itself or by close neighbours) may only be known by investigators working in the field and should also be mentioned. Of course, population names may be more difficult to assign in the case of samples of donors or patients. Then personal comments from the principal investigator are welcome. In any case, HLA-NET recommends avoiding outdated racial names like ‘Caucasian’, ‘Black’, ‘Mongoloid’ and their derivatives (see above).
3. The geographic location of the population: this has to be filled in detail (including latitude and longitude). A crucial issue is to know whether a population has been sampled in its ‘original’ location or not (e.g. Chinese living abroad). Of course this ‘original’ homeland may be traced back to only one or to many generations (e.g. back to the 15th century for Americans of European descent, etc.). Detailed information has to be provided in complicated cases.
4. The language spoken by the population: this should be filled with the help of the *Ethnologue* (http://www.ethnologue.com). Some redundancy may appear with the name of the population (see point 2 above), but here crucial information is required concerning the linguistic family.

The same questionnaire then asks information on the source of DNA samples and HLA typing, and on basic ethical issues. Detailed comments on these aspects will be found below in chapters WG2 – HLA typing standards for population genetics analyses and WG4 – Ethical issues. A delicate question is that of the number of individuals tested. We previously proposed a minimal threshold of 100 individuals (Sanchez-Mazas, 2002) and minimal sample sizes should be kept as close as possible to this threshold. Note, however, that more individuals per sample will allow detecting more alleles (eventually new ones) and will provide much better frequency estimates.

#### Collection of population data

A final objective of HLA-NET is to create a consensual map of the HLA molecular diversity of European populations in a broad sense. The population data to include as part of the HLA-NET project thus concern in priority:

1. European populations;
2. Populations from surrounding areas, i.e. North Africa, West Asia, Near-East;
3. Populations from other regions of the world but related to Europe, i.e. local minorities of European countries such as Congolese in Belgium, etc.

However, HLA-NET is closely related to other projects conducted at the international level, like the ‘Analysis of HLA Population Data (*AHPD*)’ project of the 15^th^ (Nunes *et al.*, 2010) and 16^th^ International Histocompatibility and Immunogenetics Workshop, where populations from all continents are investigated with the aim to reconstruct human peopling history. Therefore, population samples from all regions of the world may be considered by HLA-NET for further collaborations.

A preliminary list of laboratories participating in the Action and providing population or registry samples was created on the HLA-NET website through a wiki for continuous updating. The project started with a total of 14 European samples: Austrian, Belgian, Bulgarian, Bulgarian Gipsy, Croatian, Finnish, French, Greek, Italian, Norwegian, Norwegian Sami, Portuguese, Slovenian and Swiss (Table 1). Updates of the list will be found at http://hla-net.eu. Last but not least, the group benefited from the help of the European Federation for Immunogenetics (EFI, http://www.efiweb.eu/) to call for participation by using its services (mailing list, EFI newsletter) and by inviting HLA-NET to organize special sessions during its annual conferences (Florence, May 2010; Prague, May 2011).

**Table 1:** Preliminary list of population/registry samples available for HLA-NET

Name	Population	Resolution	Reporting results	Technique	SBT class I	SBT class II
G. Fischer	Austrian (registry)	Intermediate	List of ambiguities	SSO, SSP	n.a.	n.a.
M. Toungouz Nevessignsky	Belgian (registry)	Intermediate	National Marrow Donor Program (NMDP) codes	SSO,SSP,SBT	n.a.	n.a.
M. Ivanova	Bulgarian, Bulgarian Gipsy	High	List of ambiguities	SBT, SSO	Exons 2-4, biallelic	Exon 2, biallelic
Z. Grubic	Croatian	High	No ambiguities	SSO, SSP	n.a.	n.a.
M.L. Lokki	Finnish	High	List of ambiguities	SBT	n.a.	Exon 2, biallelic
V. Dubois	French (registry)	Intermediate	NMDP codes	SBT	Biallelic	Biallelic
C. Papasteriades	Greek	High	No ambiguities	SSP, SSO	n.a.	n.a.
F. Poli	Italian	High	No ambiguities	SSP,SSO,SBT	Exons 2-4, monoallelic	Exons 2-3 biallelic
B. Lie	Norwegian, Norwegian Sami	Intermediate	NMDP codes	SSP, SSO	n.a.	n.a.
D. Ligeiro	Portuguese (registry)	Intermediate	List of ambiguities	SBT, SSO	Exons 2-4	Exons 2-3
B. Vidan-Jeras	Slovenian	High	List of ambiguities	SBT, SSP	Exons 2-4, biallelic	Exons 2-3, biallelic
J.M. Tiercy	Swiss (registry)	Intermediate	List of ambiguities	SSO, SSP	n.a.	n.a.

n.a.: not applicable.

## WG2 – HLA typing standards for population genetics analyses

### Aims of group

A major aim of Working Group 2 (WG2) is to define standards for producing high-quality data for HLA genotyping and set up criteria for typing methods used for each population, thus allowing population comparisons in meta-analyses. These tasks involve careful comparisons of genetic typing methodologies and their ability to produce results at comparable resolution levels; they also address the search for strategies to handle ambiguous data and interpret heterogeneous HLA genotypes because of the very high level of complexity of this polymorphism and the adoption of universal and user-friendly formats.

#### Reporting typing ambiguities

The group worked on the issue of reporting typing ambiguities in a format that is best suitable for haplotypic and allelic frequency estimation, intra- and inter-population genetics analyses.

While the gold standard is exon 2 + 3 (class I) and exon 2 (class II) sequencing, populations may be analysed by other methods, such as reverse SSO hybridization on microbeads arrays (luminex technology). This latter method also targets exons 2 + 3 (class I) and exon 2 (class II) polymorphisms, although it can be extended to type for exons 4-7. It is ideally suited for typing large numbers of samples, but it leads to typing ambiguities in most cases, because of the ever increasing allelic polymorphism. Similarly, bi-allelic sequencing also leads to ambiguities that may be resolved using additional primers for the sequencing reactions when polymorphisms are located within the amplicon (Voorter *et al.*, 2007). Ambiguities involving polymorphisms located outside exons 2 + 3 (class I) or exon 2 (class II) require longer range PCR and additional sequencing reactions. Whatever the technique used it is recommended that all the ambiguities are reported. This is generally achieved using the National Marrow Donor Program (NMDP) coding system, i.e. abbreviation codes for the so-called ambiguous allele groups. Although certainly helpful for its original purpose of simplifying the identification of matched bone marrow donors, its use in practice increases artificially the number of allele pairs for a given genotype prior to haplotypic and/or allelic frequency estimation. To retain maximal information, it is strongly recommended to provide the list of allele pairs required to explain the genotype, as this will not include spurious allele pairs resulting from the expansion of the abbreviation codes.

**Figure 1:**
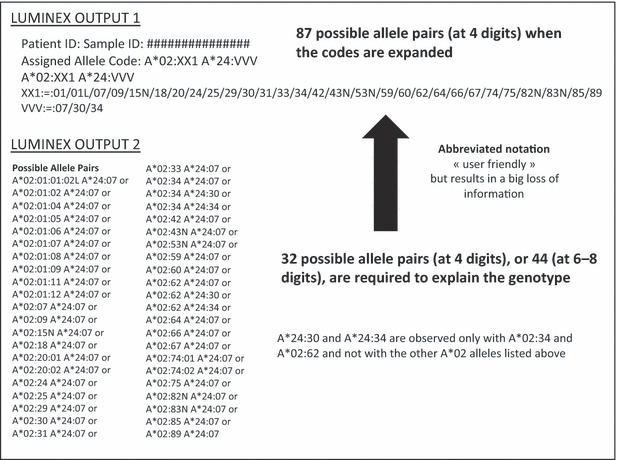
Illustration of the importance of defining an adequate and standard notation procedure for ambiguities in two alternative outputs proposed by the reverse SSO microbead array typing method.

An example of the importance of defining an adequate ambiguity notation as a standard procedure is provided in Figure 1 for two alternative outputs proposed by the reverse SSO microbead array typing. Based on the above considerations, guidelines and recommendations of WG2 for reporting HLA typing ambiguities are given in Appendix 2 and can be found at http://hla-net.eu/reporting_HLA_typings_guidelines.

Based on the IMGT/HLA database, a list of ambiguities that comprise polymorphisms outside exons 2 + 3 for class I and exon 2 for class II at each HLA locus has been generated. In a second step each of these alleles differing outside the sequence defining the peptide-binding site was screened for its occurrence in available population databases. While a majority of these belonged to the rare or very rare allele groups, several alleles were identified as occurring at significant frequencies in different populations. A list of such alleles is shown in Table 2.

**Table 2:** List of alleles (nonexhaustive) that were usually not taken into account in the past but may affect population genetic statistics because of significant frequencies

Allele	Populations
A*24:02:01:02L	Pan-European/West Asian
B*07:06	Pan-European
B*44:27	Pan-European
C*04:09N	Pan-European
C*07:06	Pan-European/West Asian
C*07:18	Pan-European/Chilean
DRB1*14:54	All populations
DQB1*02:02	All populations
DQB1*03:19	Pan-European

Whether the discrimination of these alleles has an input on population comparisons remains to be elucidated. Some data are already available showing that the relative frequencies of the DRB1*14:01 and 14:54 alleles differ widely among populations, with the DRB1*14:01 extremely uncommon in American populations from Asian descent but more frequent (up to 15%) in spanish speaking American populations (Xiao *et al.*, 2009). In Europe a recent survey of 106 German donors with DRB1*14:01/14:54 ambiguous typings found 87.9% to be DRB1*14:54 (Furst *et al.*, 2010).

#### Reporting rare alleles

As a 15th IHIW project, data were collected on the frequency of supposedly rare HLA alleles, and the final analysis showed that 40.8% of the 2977 HLA alleles (Release 2.23.00, Oct. 2008) have been sequenced only once and should therefore be considered as very rare (Middleton *et al.*, 2009). In a previous ASHI study, 27-30% of the HLA-A, B, C, DRB1 alleles have been classified as common (frequency >0.0001) or well-documented (observed at least three times) (Cano *et al.*, 2007).

Through HLA-NET, rare alleles have been submitted to Derek Middleton and Faviel Gonzalez-Galarza and included in the http://www.allelefrequencies.net database (Gonzalez-Galarza *et al.*, 2011). In total, 193 distinct alleles have been submitted (Table 3) and 70 of these submissions allowed the confirmation of an allele which had never been reported after its initial submission to IMGT/HLA (Robinson *et al.*, 2003).

**Table 3:** Rare alleles contibuted to the http://www.allelefrequencies.net database

Name	City	Country	Sent	Distinct alleles to the lab	Method(s) used
B. Lie	Oslo	Norway	5	5	SSP
B. Vidan-Jeras	Ljubljana	Slovenia	4	3	SBT, SSP
C. Papasteriades	Athens	Greece	7	3	SSP
D. Ligeiro	Lisbon	Portugal	27	27	SBT, SSP
F. Poli	Milan	Italy	38	30	SBT, SSP
G. Fischer	Vienna	Austria	26	26	SBT
J.-M. Tiercy	Geneva	Switzerland	1	1	SBT
M.-L. Lokki	Helsinki	Finland	3	3	SBT
M. Ivanova	Sofia	Bulgaria	6	6	SBT
F. Claas, D. Roelen, W. Verduijn	Leiden	Netherlands	76	66	SBT
V. Dubois	Lyon	France	50	45	SBT
Z. Grubic	Zagreb	Croatia	3	3	SSP, Other
Total			246	218*	

* Taking into consideration all submissions, 193 distinct alleles were submitted.

#### Available population samples

A list of available HLA-typed European population samples has been provided by WG2 members and will be available for the project. As shown in Table 1, 14 different populations were initially provided, with various typing techniques and number of individuals, but with high resolution typing for most populations. The list is currently being updated (see chapter WG1 – Population definitions and sampling strategies for population genetic analyses, point 2: Collection of population data).

## WG3 – Bioinformatic strategies for HLA population data storage and analysis

### Aims of group

The complexity of the HLA polymorphism is due to the existence of hundreds or thousands of different alleles at various loci and because new alleles are constantly discovered. As a consequence, HLA population data are neither stored nor analysed in a standard way in different laboratories, which makes comparisons very difficult. To use the large amount of data produced by different laboratories, in an optimized and comparable way, public access to specific computer facilities, continuously updated in relation to the ever-increasing HLA allelic record and new developments in data analysis, is required. Working group 3 (WG3) was charged of two types of tasks: first, to provide the computer infrastructure to HLA-NET and the minimal tools required to support the work of the other working groups and second, to develop the databases and computer tools required for storing and analysing the HLA data, in particular the statistical methods and computer programs necessary to validate and report data with the highest level of reliability.

#### HLA-NET infrastructure

The website of HLA-NET (http://hla-net.eu) is a *wiki* that is used to support all activities, e.g. scheduling meetings, reporting results, publishing documents, providing access to computer programs and disseminating information, among others. The *wiki* simplifies the participation of HLA-NET members to the project, making coordination possible for both small and large contributions, such as correcting typos or creating new sections of the site, respectively. In a further step, this integrated web platform will be connected to the databases that Derek Middleton and Alicia Sanchez–Mazas’ groups are currently harmonizing (Gonzalez- Galarza *et al.*, 2011; Vangenot, C., Weber, O. S., Sanchez-Mazas, A. & Nunes, J. M. In prep.). This harmonization is conceived in a way to include a number of computer programs for routine validations and analyses of HLA and other immunogenetics data. In this way, new data implemented in the future will be automatically processed according to HLA-NET standard recommendations. To understand such recommendations, we review below some crucial questions that we had to face in this part of the project.

#### Dealing with heterogeneous, ambiguous and low sample-sized data

The data collections are of diverse types, i.e. both frequency and genotypic data, and the level of resolution is quite diverse. We believe that to maintain an acceptable balance between financial cost and precision of typings, most laboratories will continue to type HLA at intermediate resolution levels including ambiguities, at least until next generation sequencing is routinely used. Therefore, considerations related to the treatment of ambiguous data are not only of interest in the present but also in the foreseeable future.

One aspect of this issue relates to the above-mentioned standardization and reporting HLA typing results (including the identification of kits, potentially typed and untyped alleles, and possible ambiguities), which is mainly a scientific task of WG2. Here, the role of WG3 is to provide computer facilities for the application of the corresponding recommendations. The programs being set up by WG3 are built on the Gene[rate] tools (http://geneva.unige.ch/generate/), the formal specifications of which have been published by Nunes *et al.* (2010, 2011b). At this level, two Gene[rate] programs are particularly useful:

1. *phenotype* to interpret raw phenotypes based on a given reactivity data file and a kit description file; and
2. *transliterate* to perform allele substitutions (e.g. to recode 2nd field (protein level, formerly 4-digit) alleles into 1st field (allele group level, formerly 2-digit) alleles) within a given dataset.

Another tool, *uniformate*, allows one to check the validity of the data format before using any other Gene[rate] tool.

In this vein, some work has been devoted to the adaptation of input formats to the guidelines recommended by WG2, and this adaptation now allows running programs for standard one-locus analyses (frequency estimation, Hardy–Weinberg equilibrium and neutrality testing). On the other hand, the feasibility of a fully automatic recoding (through the Gene [rate]*transliterate* tool) of more complex (i.e. multilocus) datasets is still progressing, as it faces the problem of the identification of the potentially typed and untyped alleles at each locus and their combination across distinct loci, as well as the standardization of the procedure across multiple samples during the same run.

An aspect of this issue relates to the use of heterogeneous and/or ambiguous data. Distinct samples collected at different times or typed with distinct techniques will not allow detecting the same alleles or specificities. Thus, to compare samples of distinct sources, the first step is to define the common set of alleles over which to work. For each allele, we face two extreme situations: the use of the unspecific first field (formerly 2-digit) allele group and the use of the precise allele defined at the highest resolution. Careful scrutiny of the data generally provides an intermediate solution where the most common allele pool between several samples includes both ‘broad’ lineages for some alleles and highly precise definitions for others. Actually, within the framework of a HLA-NET-related research project, we produced a set of programs (‘Split-test’) that provide help in screening the raw data and setting the common allele pool of a collection of samples. Recently, this ‘broad-split’ computer tool has been applied successfully to study the HLA molecular diversity of the Swiss bone marrow donors’ registrees (Buhler, S., Nunes, J. M., Nicoloso, G., Tiercy, J.-M. & Sanchez-Mazas, A. Submitted).

A challenge of this kind of work is to use information as detailed as possible at the allelic level without compromising statistical power and without making too many false-positive identifications. These two problems have become significant with the advent of high-resolution typing because in this case, the vast majority of the samples tested are too small in size to allow an accurate identification of all existing alleles. This situation is even worse when typing ambiguities are taken into account (see discussion on the use of NMDP codes on paragraph 2.1). In this context, WG3 is thus also tackling the important issue of ‘sample size and number of alleles’. We are currently adapting a tool that will make easy to estimate sample size thresholds and which will complete the efforts of WG1 working on population sampling. It is worth stressing that in general low allele or haplotype frequencies are poorly estimated when sample sizes are small and should be considered with caution. Even detected alleles may actually be ‘nonsignificant’ from a statistical point of view, depending on the sample size. A very rough number for the minimal frequency of a ‘significant allele’ is given by the confidence interval of the allele frequency obtained by normal (either two-tail or one-tail) approximation (which is a standard statistical practice) or binomial distribution. For instance, for a sample size of 50 individuals, all frequency estimates smaller than 3.85% are ‘nonsignificant’ frequencies, i.e. not significantly different from zero, because zero is inside the two standard deviations’ confidence interval (Table 4); in the same way, a 1% frequency is only ‘significant’ for a sample larger than 200 individuals. Therefore, because of the existent sampling conditions where low sample sizes are usually the rule, HLA-NET strongly recommends to avoid discussion on the ‘number of alleles present’ or ‘the presence or absence of given alleles’ in the populations where the samples were drawn. Also, rather than to fix a minimal sample-size threshold, a reasonable advice that can be given is to use samples as large as possible.

**Table 4:** Allele frequency thresholds (in %) below which the 95% confidence interval contains 0, as a function of sample size (N) and sampling model: I) standard normal two-tail; II) normal one-tail; III) exact binomial. Alleles exhibiting these and smaller allelic frequencies have probabilities larger than the usual 5% of being missed (0 alleles) in samples of the corresponding sizes

Allele frequencies			

*N*	Model I (%)	Model II (%)	Model III (%)
30	6.25	4.43	4.85
50	3.85	2.75	2.95
100	1.96	1.37	1.48
150	1.32	0.92	1.00
200	0.99	0.69	0.75
500	0.39	0.28	0.30

N, number of individuals in the sample.

#### Population genetics with ambiguous data

Having mentioned the efforts to determine the actual allele pool that can be used in a study, we now briefly report the adaptation of the population genetic methods used for routine analyses. The former (15th) IHIW workshop’s *AHPD* project held in Brazil in 2008 provided the framework to develop and test the Gene[rate] tools and their adequacy to the treatment of ambiguous data, and these tools were further expanded and generalized within the context of HLA-NET. Besides their specific abstractions (data structures) used to capture ambiguous genetic data and the definition of probability vectors to represent each individual’s data, the main characterization of these programs is the use of resampling schemas to identify the sampling distribution of each statistic (e.g. homozygosity and linkage disequilibrium) of interest (Nunes *et al.*, 2011b). Currently, it is possible to estimate allele frequencies, report frequencies graphically in the form of bar charts with colour codes, test for Hardy–Weinberg equilibrium and test for selective neutrality on data containing any number and kind of ambiguities (of course, if there are too many ambiguities the results may be meaningless but that can generally be controlled) by using the *frequency estimation* Gene[rate] tool (and *haplotype* to estimate haplotype frequencies on multiple loci). Two other programs are very useful in this context: *file conversion* allows one to convert a file into different formats (e.g. from *Excel* to the *uniformate* format used by Gene[rate]), and, as described above, *uniformate* allows one to check the validity of the data format before using any other Gene[rate] tool. All details are given by Nunes *et al.* (2010, 2011b) and the programs are available at http://geneva.unige.ch/generate.

#### Practical issues for population analyses

Although not yet definitive (ongoing work), the following HLA-NET WG3 recommendations correspond, to our view, to the most important aspects of a population analysis:

1. Genotypic data for given population samples (either anthropologically defined or registry data) should be complete and include all ambiguities; the format used should be well known or explicitly described (e.g. *uniformat*); NMDP codes should be avoided.
2. Data used for analyses should be retrieved from genotypic data by recoding distinct sets of alleles depending on the allelic pool of interest for a given analysis (e.g. by using Gene[rate]*transliterate* tool).
3. Sample sizes and the corresponding significant levels of allele frequencies (based on standard deviations) should be stated; the interpretation of the frequencies should take into account these significances and should avoid comparisons of populations based on the presence or absence of low-frequency alleles.
4. Reports of allele or haplotype frequencies should mention the program and, possibly, the algorithm used for estimation. Ideally, details about the initial conditions and environment of the algorithm used should also be included (e.g. for an expectation-maximization (EM) algorithm: the number of starting points, the number of distinct solutions, and the convergence criteria, i.e. either on likelihood or frequency values).
5. Assessment of Hardy–Weinberg equilibrium (HWE) is mandatory for any use of allelic frequencies describing the genetic profile of a population in comparison to other data (otherwise phenotypic frequencies should be used). Testing for HWE using chi-square, G or exact tests on contingency tables should only be done in the absence of ambiguities and blank-like alleles. Otherwise, methods explicitly accommodating ambiguities should be used, like the method using nested likelihood ratios implemented in the Gene[rate]*frequency estimation* program.
6. Selective neutrality should be assessed at least by reporting expected and observed homozygoties; a formal test (e.g. the Gene[rate] algorithm of Ewens–Watterson test implemented in the *frequency estimation* program) is however preferable.
7. One should use bar-chart graphics to represent frequencies, rather than pie charts that are prone to many errors for comparisons (see Tufte, 2001).
8. Proper studies should also include an account of ethics as per WG4 recommendations.

An experiment is currently being made to accommodate this kind of meta-information described above in the context of the *AHPD* project of the 16^th^ IHIW workshop. The WG3 group will evaluate the results afterwards.

The issues mentioned above show that WG3 is fulfilling its goals by providing a fully operational implementation of the guidelines emerging from HLA-NET. Furthermore, given that the Gene[rate] programs are formally described, it will be easy to implement them in other platforms developed for population genetic analysis. The applicability of WG3 work thus extends beyond its current Gene[rate] implementation in the HLA-NET platform.

## WG4 – Ethical issues

### Aims of group

The role of working group 4 (WG4) is to provide support to the other working groups such that their actions are undertaken with sound ethical and legal considerations. Much work has already been undertaken to address ethical issues relating to genetic analyses taking into account the interests of all the parties involved in the study, i.e. researchers, participants and society (Deschênes *et al.*; Robertson, 2003). It must be stressed that population analysis of HLA types is not equivalent to genetic screening for a mutation predictive of disease and therefore the outcome of the HLA analysis is less likely to have any impact on the participants donating to the study.

It is not the aim of WG4 to reproduce (inter)national legislation and professional recommendations that have been made elsewhere (Laberge, 2003) but to look at the application of such recommendations to HLA typing population studies specifically. In achieving our goals we aim to gather information related to legal and ethical regulations in different countries and to compile the information gained to obtain a consensus on practice for European countries.

### Study plan

Overall, a thorough ‘study plan’ is key to the success of any HLA population study, and this care must be taken to ensure that the study conforms to national and international legislation or recommendations/guidelines, where legislation is not in existence.

The study plan must be produced to provide information required for approval by institutional review board (IRB) or ethics committee. Even if the study is already covered by existing ethics approval, it is recommended that complete documentation of the study plan is produced.

The study plan must address the other following aspects:

#### Study aims

The aim of the population genetics study must be well defined and documented prior to the study taking place, i.e. which population will be studied and which genetic loci will be analysed.

#### Sampling

The following questions should be addressed:

1. Are there any risks and/or benefits to the subjects participating in the study?
2. Is the collection of new samples required or will DNA or other biological material already collected be used?
3. Will the samples be anonymized? If yes, will this happen at the point of collection or afterwards and will the link between subject and sample be reversible or irreversible. If reversible (also referred to as ‘identifiable’, ‘linked’ and ‘coded’), who is responsible for the linking information?
4. How and where will linking information be stored?

Ethical issues relating to sampling individuals and populations for genetic analysis have been reviewed elsewhere and these apply to population studies of HLA (Godard *et al.*, 2003).

### Samples already in collection

For samples that have already been collected, the consent given at the time of collection must be reviewed to see if the new proposed study qualifies. For example, samples taken for clinical testing are unlikely to have consent for unrelated HLA typing studies. Depending on what consent has been given it may be necessary to obtain additional consent and/or IRB/ethics committee approval for the HLA population genetics study. It is important to know whether the samples can be identified or not. A recent case in the USA highlighted that usage of previously collected DNA from an Amerindian tribe was not undertaken with appropriate consent from the participants (Couzin-Frankel, 2010).

### Samples to be collected

Informed consent is required and ethical committee approval must be sought. For informed consent to be given, subjects must be deemed as competent to give consent. Consent may be taken verbally or in writing depending on local legislation and guidelines but in all cases must be documented by the investigator.

The consent process must inform the subject, usually through the issuing of an information sheet, of the following (McGuire & Beskow, 2010):

1. the nature and goals of the research study
2. the type of sample to be taken from the participant
3. what sort of tests will be performed on their sample
4. whether the samples are to be made available for future undetermined studies
5. how data will be shared
6. what samples will be stored (intact cells, DNA)
7. length of storage, will this be limited
8. whether samples will be anonymized
9. freedom to withdraw from the study at any time
10. potential benefit or lack of benefit to participant
11. whether samples may be made available for other ethically approved studies

If the collection of material is from a well-defined population, it is appropriate to gain consent from appropriate authoritative members of the community and involve public consultation making use of local media prior to embarking on collection.

Successful sample collection requires the concomitant gathering of predetermined subject information (e.g. demographic and clinical data). The format of the data to be collected for each subject donating a sample must be predetermined and compatible with international nomenclature and downstream data analysers.

#### Examination process including data analysis

All biological material donated for research is extremely valuable and maximum effort must be taken to ensure that the material is tested by optimum procedures that will ensure maximum benefit from the data generated. Therefore the study plan must consider the methods that will be utilized and whether these methods will be undertaken by qualified and experienced personnel, e.g. HLA typing to be undertaken by an EFI/ASHI-accredited laboratory that participates in appropriate external proficiency testing for HLA typing. As the number of HLA alleles continues to increase with time, all HLA typing population studies must record the HLA allele database that has been used to assign HLA types to subjects such that future analysis can be undertaken should a new allele be found that may have been masked by previous typings.

The analysis of the data must also be undertaken using secure and proven software and should include application of Hardy–Weinberg.

Decisions must be taken at the time of study design to determine when resampling and/or retesting samples would be necessary.

For HLA typing data to accurately reflect the population under study, care is required to minimize unknown analysis of samples from individuals that are related to one another; this may be more difficult to determine for samples that are already in collection and therefore the numbers of samples to be analysed must be taken into consideration depending on whether knowledge is available on relatedness within population for optimum statistical evaluation.

#### Data sharing

Consideration must be given to the following:

1. Data sharing with not-for-profit and for-profit organizations. Control of who has access to the data is irrelevant if the data are made available via open access data sharing. There is always the possibility that the data obtained from the study could be used to ultimately provide financial benefit, e.g. use of HLA population data by commercial companies. A risk assessment regarding this should be made for each study and appropriate information given in the study information guide given to participants.
2. It is also important to determine prior to embarking on the sample collection whether the data should be shared with the participants. If the samples are to be anonymized then this is not possible and participants should understand this (Hull *et al.*, 2008). The issue over whether it is ethical to deny genetic research participant-individualized results have been discussed by others (Affleck, 2009). If the sharing of research results with participants is to be undertaken this could be via a newsletter or a website. This would allow a continued relationship with the participants which may be important should subsequent research studies be proposed with the participants samples (Beskow & Smolek, 2009).
3. If samples are not fully anonymized, the identifiable material/data must be kept in a secure location by the principle investigator only. Coded data should only be shared.

#### Sample handling and sample and data storage

It is crucial that sufficient finances are available to cover secure storage of samples and data and that it is clearly defined who has responsibility for samples, their derivatives and the data generated.

There must also be secure procedures in place to allow monitoring of the movement of data and samples (Godard *et al.*, 2003).

## Conclusion and perspectives

In this study, each working group has made a number of suggestions that can be taken as consensual *HLA-NET methodological recommendations*. These preliminary recommendations will of course be refined during the last period of the Action until their final publication. Compared to other proposals aiming at normalizing methodological issues in immunogenetic studies involving HLA data (Hollenbach *et al.*, 2011; Nunes *et al.*, 2011a), the present HLA-NET guidelines are the results of a large collaborative effort aiming at coordinating the whole suite of steps necessary to analyse HLA molecular data in human populations or registries, i.e. from population and/or sample definition (WG1) to ethical considerations (WG4) via the reporting of typing results (WG2) and the statistical analysis of the data (WG3). Also, it proposes very concrete and immediately applicable solutions to common problems (e.g. formatting data, estimating frequencies with ambiguities) by opening the access to user-friendly and continuously developing computer tools (Gene[rate]) to the whole community of researchers working with this kind of data either at the population or at the donor-registry level. Overall, following the HLA-NET methodological recommendations given in this study will help to synchronize the work done by different laboratories to obtain comparable data and facilitate both European and international collaboration in histocompatibility, clinical transplantation, epidemiology and population genetics. At the end of the HLA-NET Action, all final documents and guidelines will be uploaded on a user-friendly HLA-NET public platform, which will also offer direct access to databases linked to useful computer programs for HLA data analysis. A joint effort with other consortiums will further be undertaken to provide widely consensual solutions at the international level.

## Appendix 2 guidelines for reporting HLA typings

### Typing Resolution

Whenever possible, perform **allelic or high resolution** typing following EFI standard D1.320 and the document ‘HARMONISATION OF DEFINITIONS OF HISTOCOMPATIBILITY TYPING TERMS’ (see http://hla.alleles.org).

a. Allelic resolution is a DNA-based typing result consistent with a single allele as defined in a given version of the WHO HLA Nomenclature Report.

b. High resolution is defined as a set of alleles that specify and encode the same protein sequence for the peptide binding region of an HLA molecule and that excludes alleles that are not expressed as cell-surface proteins. It identifies HLA alleles at the resolution level of the 2nd field (formerly 4-digit) or more, at least resolving all ambiguities resulting from polymorphisms located within exons 2 and 3 for class I loci, and exon 2 for class II loci.

c. Intermediate resolution is defined as a DNA-based typing result that includes a subset of alleles sharing the digits in the first field of their allele name and that excludes some alleles sharing this field.

d. Low resolution is a DNA-based typing result at the level of the first field (formerly 2-digit) in the DNA-based nomenclature. If none of the above resolutions can be achieved, DNA-based **low resolution** typings are accepted.

### Data with Ambiguities/high or Intermediate Resolution

In case allelic resolution is not achieved, **data with ambiguities** are accepted in the following formats (in preferential order):

- **List of possible genotypes (i.e. pairwise allelic combinations)** e.g. B*08:01:01G,B*15:18:01 or B*08:21,B*15:93 or B*08:35,B*15:10:01 (corresponding to 3 possible combinations)
- **Allelic strings** e.g. B*08:01/21/35,B*15:10/18/ 93 (corresponding to 9 possible combinations)
- **NMDP codes** e.g. B*08:MDY,B*15:DZBP (corresponding to 9 possible combinations)
